# A Quantitative and Dynamic Model of the Arabidopsis Flowering Time Gene Regulatory Network

**DOI:** 10.1371/journal.pone.0116973

**Published:** 2015-02-26

**Authors:** Felipe Leal Valentim, Simon van Mourik, David Posé, Min C. Kim, Markus Schmid, Roeland C. H. J. van Ham, Marco Busscher, Gabino F. Sanchez-Perez, Jaap Molenaar, Gerco C. Angenent, Richard G. H. Immink, Aalt D. J. van Dijk

**Affiliations:** 1 Bioscience, Plant Research International, Wageningen UR, Wageningen, The Netherlands; 2 Biometris, Wageningen UR, Wageningen, The Netherlands; 3 Max Planck Institute for Developmental Biology, Molecular Biology, Tübingen, Germany; 4 Laboratory of Molecular Biology, Wageningen University, Wageningen, The Netherlands; 5 Netherlands Consortium for Systems Biology, Amsterdam, The Netherlands; 6 Chair group Bioinformatics, Wageningen University, Wageningen, The Netherlands; Universidad Miguel Hernández de Elche, SPAIN

## Abstract

Various environmental signals integrate into a network of floral regulatory genes leading to the final decision on when to flower. Although a wealth of qualitative knowledge is available on how flowering time genes regulate each other, only a few studies incorporated this knowledge into predictive models. Such models are invaluable as they enable to investigate how various types of inputs are combined to give a quantitative readout. To investigate the effect of gene expression disturbances on flowering time, we developed a dynamic model for the regulation of flowering time in *Arabidopsis thaliana*. Model parameters were estimated based on expression time-courses for relevant genes, and a consistent set of flowering times for plants of various genetic backgrounds. Validation was performed by predicting changes in expression level in mutant backgrounds and comparing these predictions with independent expression data, and by comparison of predicted and experimental flowering times for several double mutants. Remarkably, the model predicts that a disturbance in a particular gene has not necessarily the largest impact on directly connected genes. For example, the model predicts that *SUPPRESSOR OF OVEREXPRESSION OF CONSTANS* (*SOC1*) mutation has a larger impact on *APETALA1* (*AP1*), which is not directly regulated by SOC1, compared to its effect on *LEAFY* (*LFY*) which is under direct control of SOC1. This was confirmed by expression data. Another model prediction involves the importance of cooperativity in the regulation of *APETALA1* (*AP1*) by LFY, a prediction supported by experimental evidence. Concluding, our model for flowering time gene regulation enables to address how different quantitative inputs are combined into one quantitative output, flowering time.

## Introduction

Flowering at the right moment is crucial for the reproductive success of flowering plants. Hence, plants have evolved genetic and molecular networks integrating various environmental cues with endogenous signals in order to flower under optimal conditions [1]. Various input signals are received and transmitted by signal transduction pathways including the photoperiod pathway, the vernalization pathway, the ambient temperature pathway and the autonomous pathway [2]. Finally, the input from these pathways is integrated by a core set of flowering time integration genes (“integration network”). This regulation contributes to the adaptation of plants to different environmental conditions and facilitated the successful dispersion of flowering plants over the world [2].

The complexity of flowering time regulation is enormous, even when focusing on the network involved in integrating the various signals. To understand how gene disturbances influence flowering time, it is not only important to know which genes regulate each other, but also how strongly these genes influence each other. Hence, quantitative aspects of flowering time changes upon perturbations of input signals cannot be understood by merely assessing qualitatively which interactions are present. To this end, a quantitative model describing how different genes in the network regulate each other is needed. Indeed, other complex plant developmental processes have been subject to extensive modeling efforts [3]. This includes processes such as the circadian clock [4–7], auxin signalling [8–11], photoperiod regulation of flowering time genes [12,13] and the development of floral organs [14–17], which all have been investigated in detail by computational models. These models enable to formalize biological knowledge and hypotheses, and, importantly, to investigate how various types of inputs are combined to give a quantitative readout.

Flowering time regulation has been extensively studied experimentally in the plant model species *Arabidopsis thaliana*. Substantial qualitative information is available about the factors involved and how these interact genetically. However, the information that is needed for quantitative and dynamic modelling is missing to a large extent. This includes comprehensive and standardized quantitative data on flowering time under various conditions and in different genetic backgrounds [18], and time series of expression for key flowering time integration genes in those backgrounds. In line with the scarcity of quantitative information useful for modelling, the floral transition in *Arabidopsis thaliana* has been scarcely studied using modeling approaches. Recently a few promising mathematical modeling approaches appeared aimed at modeling the floral transition in various plant species [19–21]. Dong et al. modeled a network of four genes involved in the floral transition in maize [19], and Satake et al. modeled a two-gene network in *Arabidopsis halleri* [21]. Earlier work on modelling *Arabidopsis thaliana* flowering time did not take genetic regulation into account or used a mainly qualitative approach [22]. Only very recently a first quantitative model of the *Arabidopsis thaliana* flowering time integration network was presented [20].

**Fig 1:**
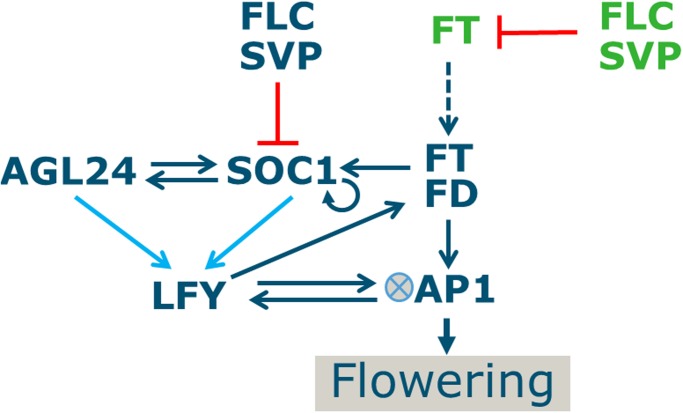
Network of flowering time integrator genes. Green indicates expression in leaf tissue, blue in meristem tissue. Red arrows represent repression, blue arrows activation. Most interactions were taken as given based on literature information, but for regulation of *LFY* by AGL24 and SOC1, different ways of combining the two inputs were tested (indicated by the light blue arrows). Dashed arrow represents FT transport. Junction symbol next to *AP1* indicates cooperativity predicted for regulation of *AP1* by LFY. As indicated, *AP1* expression is used as a marker for the moment of the floral transition. This network was used to fit expression time-course data and to predict the effect of perturbations. Gene names are given in full in the text.

We aimed to obtain a mechanistic understanding of the *Arabidopsis thaliana* flowering time integration network, by investigating a core gene regulatory network composed of eight genes (Fig. 1): *SHORT VEGETATIVE PHASE (SVP)*, *FLOWERING LOCUS C (FLC)*, *AGAMOUS-LIKE 24 (AGL24)*, *SUPPRESSOR OF OVEREXPRESSION OF CONSTANS 1 (SOC1)*, *APETALA1 (AP1)*, *FLOWERING LOCUS T (FT)*, *LEAFY (LFY)* and *FD*. Although certainly more genes are involved in integrating the various signals influencing the timing of the floral transition [2,23], we focussed on these genes because a) we aim to model the core of the network responsible for flowering time regulation; and b) for these genes, the available experimental data renders a clear picture of their mutual interactions (see above; Table A in S1 File; Fig. 1). In the leaves, SVP and FLC repress the transcription of *FT* [24–27]. FT is produced in the leaves and moves to the shoot apical meristem (SAM) [28,29]. FT has the potential to interact with FD [30,31] and complex formation is supposed to occur at the SAM, leading to activation of *SOC1* [32] and *AP1* expression [30,33]. *FLC* and *SVP* are also expressed in the SAM, where they repress the expression of *SOC1* [34–36]. *SOC1* integrates signals from multiple pathways and transmits the outcome to *LFY* [37,38], which is supposed to act at least partially via a positive feed-back loop in which *AGL24* is involved upon dimerizing with SOC1 [39]. In turn, LFY is a positive regulator of *AP1* [40] and of FD [20]. The commitment to flower is ascertained by a direct positive feed-back interaction between *AP1* and *LFY*. Once the expression of *AP1* is initiated, this transcription factor orchestrates the floral transition by specifying floral meristem identity and regulating the expression of genes involved in flower development [41]. Importantly, in comparison with the recently presented model of the floral transition in Arabidopsis [20] we included the key floral integrator genes *SOC1*, *SVP* and *AGL24* in our model.

The above introduced interactions between the flowering time integration genes and the floral meristem identity genes at the end of the pathway allow to derive a set of Ordinary Differential Equations (ODEs) describing how genes in the network regulate each other. ODEs were chosen because they arise from continuum modelling of molecular interactions and allow quantitative analysis of the effect of perturbations on expression levels and finally on flowering time. Because of the above mentioned role of *AP1* as orchestrator of floral meristem identity specification, the moment at which the *AP1* expression level starts to rise is used as a proxy for flowering time in the model.

In order to build and validate an ODE model describing the network constituted by the eight selected genes, we obtained three quantitative datasets: i) gene expression time-courses of the selected eight genes in wild type; ii) flowering time of plants of different genetic backgrounds; and iii) expression data of the selected genes in the plants of these different genetic backgrounds. A key aspect of our approach is that we estimate model parameters using the dynamic gene expression time-course data for the components of the model, in combination with flowering time data (datasets *i* and *ii*). We validated our model by comparing predicted expression time-courses for mutants in components of the network with experimental data (dataset *iii*). Finally, we obtained detailed understanding of how genes are affected by perturbation in other genes, via the regulatory interactions that constitute the network.

## Results

### Model building and parameter estimation

Given the importance of combining various input signals into a final decision to flower, a key question is how the integration network generates a quantitative response, i.e. how expression level perturbations of various magnitudes result in specified changes in other network components and finally in a change in flowering time. In order for the model to be able to link expression changes to changes in flowering time, we included *AP1*: expression of *AP1* indicates that the switch from vegetative to reproductive growth has occurred [42]. As such, we use the moment at which *AP1* expression rises above a certain threshold in our model as a proxy for the moment at which flowering starts (see Methods for details).

Our approach to investigate the network involves modelling by ordinary differential equations (ODEs), which describe how the expression level of each gene is influenced by the other genes. This regulation is described by Hill functions [43], which represent activation or repression by the various regulators. Genetic and molecular knowledge on the network structure is used as input to define these equations. Parameters in these equations represent interaction strengths and other biological or physical aspects of the system, and are estimated using wild-type gene expression time-course data. *FLC* and *SVP* are not known to be regulated by any of the genes included in our model, and for that reason, they are included as external input factors, that regulate one or more other genes in the model. In order to model transport of FT protein to the shoot apical meristem [44], we assumed that the FT produced in the leaves reaches the meristem with a delay. An optimal parameter set, which includes the FT transport delay, was identified by fitting the equations to qRT—PCR time-course data from leaves and SAM-enriched material obtained from Arabidopsis plants grown at 23°C under long-day (LD) conditions (Tables B-C in S1 File).

**Fig 2:**
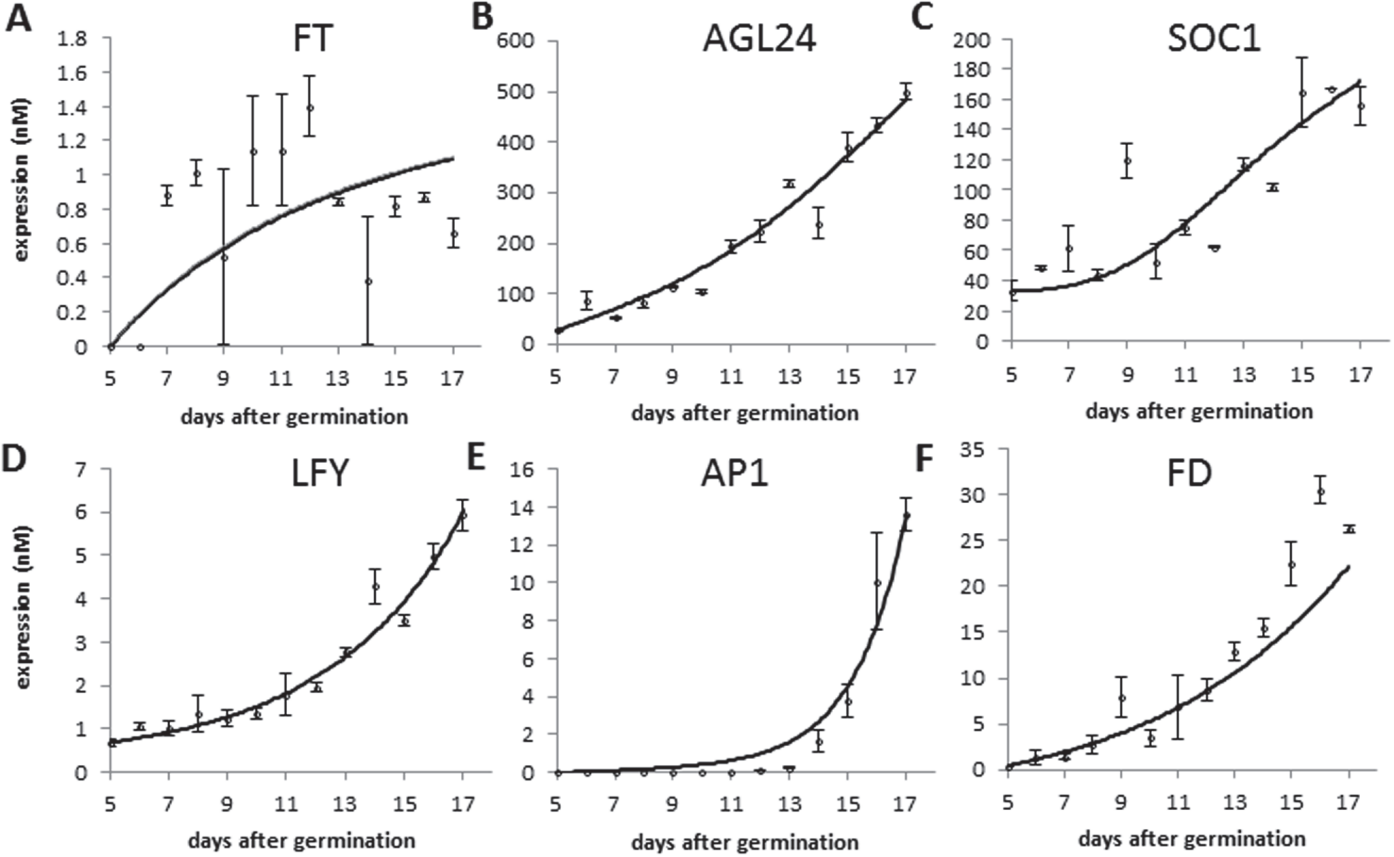
Experimental and simulated expression time-course of the genes in the integration network model. Gene expression was measured by qRT-PCR (shown as dots) of wild type plants grown under long-day conditions at 23°C (average and standard deviation are shown). The continuous lines show the simulated gene expression using the parameters estimated by data fitting. Note that *FLC* and *SVP* are not regulated by other components of the network and hence are present as input factors only, and their expression level is not simulated by the model. qRT-PCR data for *FT* was obtained from leaves; for the other genes, qRT-PCR data was obtained from meristem enriched material.

The genes in the core regulatory network of flowering time control cooperate to activate the flowering orchestrator *AP1* [41]. This allows proper timing of *AP1* expression and fine tuning of flowering time in response to different environmental cues. In wild type Arabidopsis, the *AP1* level remains barely detectable in the SAM until about day 13 after germination and then sharply increases (Fig. 2). As mentioned above, we use the moment at which *AP1* expression level rises as a marker to indicate that the transition to reproductive development is completed, which is interpreted as a predictor of flowering time. Based on that, we developed a fitting strategy that besides of aiming at a good fit, optimizes the correlation between predicted and observed flowering time. To be able to do so, we obtained a consistent set of flowering time measurements for mutants of six of the genes in our network (Figure A in S1 File). Flowering time was measured as the number of rosette leaves (RL) present at flowering. To compare model predictions for flowering time, expressed in units of days, these were scaled to RL (Methods).

A total of 35 parameters in six equations were estimated from the time series data containing 13 datapoints (expression levels) per gene (Tables B-C in S1 File; Fig. 2). Given the variability in the data, the fit is satisfactory, as indicated by the value of the normalized root mean square error (nrmse). For *FT*, for which the data shows highest variability, the highest nrmse (27%) was obtained. For *SOC1*, the overall fit was good, but does not capture the data point at day 9, which deviates from the general trend in the time series, resulting in a nrmse of 19%. For *AP1* and *FD* the value of the nrmse was around 14%, and for *AGL24* and *LFY* it was 7%. The *FLC* and *SVP* expression data were used directly as input to the model; these are shown in Figure B in S1 File. Interestingly, for the data describing *AP1*, we could only obtain a good fit by introducing a particular value of one parameter describing how *AP1* is regulated by LFY. As further discussed below, this parameter indicates DNA binding cooperativity for which indeed experimental evidence exists.

**Fig 3:**
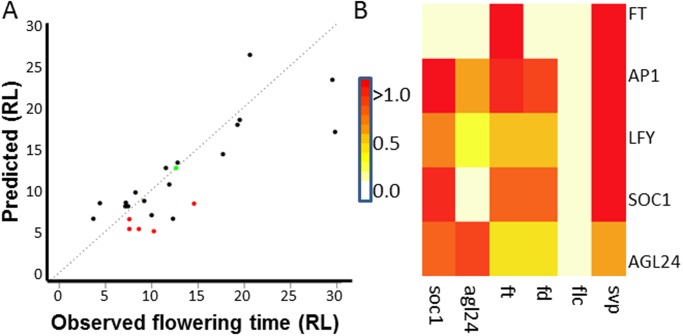
Model predictions and experiments in various mutant backgrounds. (A) Predicted vs. experimentally observed flowering time for mutants used in training the model (black) and for double mutants used for validation (red). Wild type flowering time is indicated in green. RL, rosette leaves: the more rosette leaves, the later flowering. (B) Prediction of expression changes; total change in expression over the simulated time-course is calculated, normalized against wild type; absolute value is reported to focus on the magnitude of the predicted expression change. Horizontal axis, mutants; vertical axis, genes for which expression change in mutant background is simulated. Note that FLC and SVP are not regulated by other genes in the model and hence, their expression level does not change upon any mutation. For comparison between predictions and experiments, see Figures C and D in S1 File.

Simulated flowering times in various genetic backgrounds are shown in Fig. 3A. There is one outlier in this plot (*ft-10*; observed flowering time 30 RL, predicted flowering time 17 RL). Besides this exception, there is considerable agreement between data and predictions. Indeed, comparison of the Pearson correlation (R = 0.85, including the outlier) with correlation obtained using randomized data demonstrates the significance of this result (p<0.005), indicating a satisfactory model fit.

### Model validation

A key issue in our model is the mechanism by which the network is able to give a quantitative response to specific perturbations. How are changes in a given gene expression level transferred to other components of the network, and how does this impact flowering time? In order to validate model predictions of how changes in expression propagate through the network, we simulated the expression time-courses for mutants and obtained independent experimental data for comparison. For that, microarray experiments were used, which were carried out for wild type and four mutant backgrounds (*soc1*, *agl24*, *fd* and *flc*). In these experiments, a flowering inducing shift from short-day to long-day conditions was used [45]. To account for the fact that these experimental conditions cannot directly be simulated, our comparison of the experimental microarray data with simulation results focusses on the overall effect of a mutation over the complete time-course (Methods). As indicated by the value of Pearson’s R (0.69; p-value = 0.003), the predicted overall expression level changes of flowering time genes upon upstream mutations show a significant correlation with the experimental data (Figs. C-D in S1 File). Assessing the correlation per gene (across the different mutants) indicates similar correlation for each of the genes. However, assessing the correlation per mutation (across the different genes) indicates good predictive performance for *SOC1*, *FD* and *AGL24* mutations, but not for *FLC* mutation. The latter could be due to the low expression and limited role of *FLC* in the Col-0 background due to the *FRIGIDA (FRI)* mutation [46]. The comparison with the microarray dataset constitutes an independent evaluation of the predictive performance of the model, demonstrating that the model allows predicting the overall magnitude of the effect of a perturbation in one gene on other genes in a quantitative manner.

To further assess the predictive performance of the model, we analysed five double mutants in which over-expression of one gene was combined with knock-out of a second gene. In all cases, both genes involved activators of flowering (Fig. A in S1 File), implying that it is intuitively difficult to predict whether the double mutant will be early or late flowering. These mutants were not used in the parameter estimation stage. The resulting prediction performance was satisfactory (Fig. 3A): for four out of five cases, the prediction was qualitatively correct (“early flowering”). Quantitatively, the correlation between experimental and predicted flowering times was reasonable as well, although not significant at the p = 0.05 level (Pearson R = 0.75; p = 0.1). It is good to realize that no perfect fit was expected in this case because of variable temporal and spatial overexpression levels due to the usage of the 35S promoter [47].

### Spread of perturbations through the network

As a first example of quantitative understanding of flowering time regulation, we analysed the predicted expression changes in various mutant backgrounds (Fig. 3B). A key question here is how gene expression perturbations spread through the network. We found that the model predicts that the spread of a perturbation is not in all cases directly related to the position that different genes have in the network (Fig. 3B). For example, the effect of mutating *SOC1* on *LFY* is smaller than its effect on *AP1*, although SOC1 regulates *LFY* and does not directly regulate *AP1*, but only indirectly via LFY. Analysis of the regulatory interactions and the associated parameters in the model allows rationalizing such differences. For the above-mentioned different magnitudes of the effect of *soc1* mutation on *LFY* compared to its effect on *AP1*, it is relevant that the estimated expression activation strength (parameter β) for the influence of SOC1 on *LFY* (β_7_) is much smaller than that for the influence of LFY on *AP1* (β_9_; Table C in S1 File). This means that the model predicts that a change in SOC1 will give rise to a relatively small change in *LFY*, which however will be amplified by LFY regulating *AP1*. This effect is visible in the experimental microarray data as well, where in the *soc1* mutant background *LFY* expression is much less affected than *AP1* expression (normalized *AP1* expression change in the *soc1* mutant compared to wild type is two times that of *LFY*; Figs. C-D in S1 File). This illustrates that the effect of perturbations can considerably grow in magnitude throughout the network.

### Regulation of AP1 by LFY

As mentioned above, for the regulation of *AP1* by LFY our initial analysis using the PCR time-course data indicated that we needed to introduce DNA-binding cooperativity in the equations in order to get a reasonable fit of the data. There is indeed experimental evidence for cooperativity in the LFY—*AP1* interaction, based on the LFY protein-DNA structure and additional experimental data [48]. In our modelling approach, cooperativity is defined by a Hill coefficient *n*>1 in the term in the differential equation describing the regulation of *AP1* by LFY. For the regulation of *AP1* by LFY, setting the value of *n* = 3 resulted in a markedly improved fit of the wild type time-course data (Fig. E in S1 File). No improvement of the fit could be obtained for the other interactions in the network by the introduction of a Hill coefficient larger than 1, meaning that the data does not contain evidence for cooperativity in those interactions. Cooperativity in the LFY—*AP1* interaction provides an additional predicted mechanism by which a small change in LFY, can lead to a large change in *AP1* expression. Experimental evidence indeed suggests that cooperativity in LFY binding to the AP1 promoter is important [48].

### Regulation of LFY by AGL24 and SOC1

**Fig 4:**
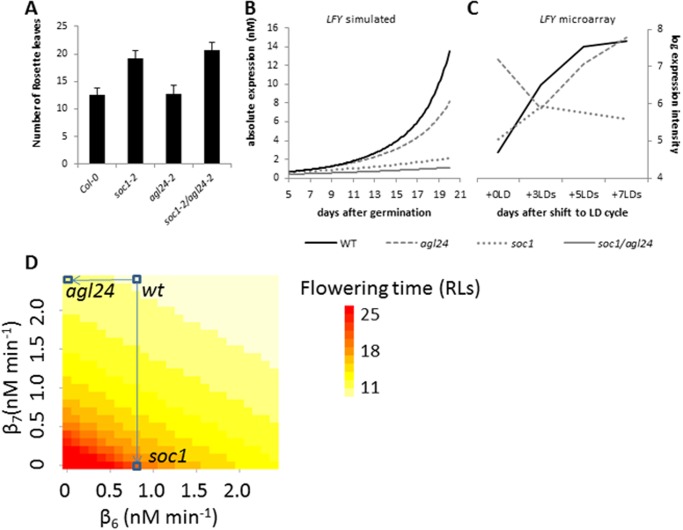
Effect of knockout mutations (agl24, soc1 and soc1/agl24) on LFY expression and on flowering time. **(A)** Number of rosette leaves counted at the onset of flowering for wild type and mutants. The plants were grown in long-day conditions at 23°C. **(B-C)** *LFY* expression in wild type and mutants from simulations (B) or microarray experiments (C). The simulations show the expression time-course over 20 days after germination; the microarray data consist of four time-points after transfer of plants grown in short-day to long-day conditions. **(D)** Effect of efficiency by which *LFY* expression is activated by AGL24 (β_6_) and SOC1 (β_7_), on predicted flowering time. Flowering time, predicted flowering time for given values of parameters. Blue boxes in heatmap indicate best-fit model parameters and the two mutants *soc1* and *agl24*; arrows point from wild type model to mutants.

It has been suggested that SOC1 requires dimerization with AGL24 for binding to the *LFY* promoter. This is based on several sources of experimental evidence: (I) in yeast-two-hybrid assay, AGL24 and SOC1 form a heterodimer [49]; (II) SOC1 is only detected in the nucleus when AGL24 is present as well [39]; (III) *LFY* is expressed only in those tissues where *SOC1* and *AGL24* expression overlap [39]. Nevertheless, there is a significant difference between the flowering time observed for *soc1* and *agl24* mutants (Fig. 4A). If these two proteins would bind the *LFY* promoter as AGL24-SOC1 dimer only, then knockout mutations in either *AGL24* or *SOC1* would equally reduce the dimer concentration; therefore, one would expect the same effect on *LFY*.

Based on these considerations, in our final model, AGL24 and SOC1 have independent roles in regulating *LFY*. We tested an alternative model version in which AGL24 and SOC1 only regulated *LFY* as a dimer and not separately from each other. This resulted in a decreased goodness-of-fit in particular for *LFY* (nrmse 43% instead of 7%) and in this alternative model, indeed the effect of *agl24* and *soc1* mutation on LFY and on flowering time were comparable, which contradicts available experimental data.

In our model, in which AGL24 and SOC1 have independent roles in regulating *LFY*, the simulated *LFY* expression is reduced by only ~25% in the *agl24* knockout mutant relative to its time-course expression in wild type. In contrast, *LFY* expression is predicted to be reduced by ~65% in the *soc1* mutant (Fig. 4B; Fig. C in S1 File). These predicted changes are consistent with what is experimentally observed in the microarray data for *LFY* (Fig. 4C). If the expression level of *SOC1* in wild type would be much higher than that of *AGL24*, a hypothesis to explain the observed difference between *agl24* and *soc1* could be that elimination of such more abundant factor would have a larger effect. However, in our expression data, expression levels of *AGL24* and *SOC1* are of the same order of magnitude. According to the model, two parameters are important in describing the regulatory effect of SOC1 and AGL24 on *LFY*: DNA binding efficiency (represented by parameter K) and expression activation strength (parameter β). A difference in any of these two parameters between SOC1 and AGL24 could lead to a difference in the effect of *SOC1* versus *AGL24* mutation. In the set of parameter values we obtained for our model, the DNA binding efficiency for AGL24 (K_10_) and SOC1 (K_11_) binding to the *LFY* promoter is quite similar. However, there is a substantial difference in activation strength (β_7_ vs β_6_), with SOC1 being much more able to activate *LFY*, resulting in a much larger effect of *soc1* mutation compared to *agl24* mutation. Analysis of predicted flowering times for a range of values of β for SOC1 and AGL24 confirms the dependency on the SOC1 activation strength (Fig. 4D). In addition, the flowering time observed for the double mutant *soc1/agl24* suggests a small additive effect when both genes are simultaneously knocked-out (Fig. 4A). The difference between the flowering time of the double mutant and that of the single *soc1* and *agl24* mutants is significant (t-test; p-value 0.001). In agreement with this observation, the model simulation predicts a small additional reduction in *LFY* expression for the *soc1/agl24* double mutant (~80% vs. ~65% in single mutant; Fig. 4B). Overall, these examples demonstrate how we get quantitative insight in the spread of perturbations through the network. Moreover, this demonstrates that we can analyse how the quantitative output of the network as a whole is governed by specific molecular interactions that build up the network.

## Discussion

An important reason to apply computational models to a biological system, such as the floral integration network, is that it allows investigating how the various interactions that together constitute the network, transmit perturbations into a final readout. Indeed, by integrating experimental data with modeling we analyse how different components of the flowering time regulation network react to changes in other components, finally leading to a specific flowering time. We specifically analysed the regulation of *LFY* by SOC1, the regulation of *LFY* by AGL24, and the regulation of *AP1* by LFY. In these cases, the activation strength was found to be the most important cause of the observed differences in magnitude of effect of perturbations, according to the model. This could mean that the protein with the higher predicted activation strength itself is a stronger transcriptional activator than the other protein, or it could indicate involvement in a protein interaction with a partner (not explicitly included in the model), which is a stronger activator. In the case of the different effect of the *soc1* mutation compared to the *agl24* mutation, it is important to consider that both SOC1 and AGL24 are known to form additional complexes, and such dimers might also play a role in their differential functioning [49]. In addition, as a general note on our interpretation of parameter values, it is important to realize that we use a fixed conversion of mRNA levels to protein levels; this means that potential differences in e.g. translation rate could complicate the interpretation of the parameters.

In a recently published review, an overview is given of attempts to model plant reproduction Gene Regulatory Networks, including networks involved in flowering time regulation [50]. Previous work on modelling flowering time used the concepts of “photothermal units” or variants thereof as a way to computationally investigate flowering time and how it is influenced by the environment; as recently demonstrated, such models can in principle be connected to genetic information [51]. However, this does not provide a direct way to incorporate the regulatory interactions between genes, which are key towards a mechanistic understanding of flowering time regulation. Our work is more comparable to recent approaches, which start with defining interactions in a gene regulatory network and then develop a model based on this network [19,20]. Our approach extends the recently published Arabidopsis flowering time model [20] by fitting model parameters using dynamic expression data. The model by Jaeger et al. predicts a rather gradual upregulation of AP1, which does not reproduce the sharp transition from low expression to the on-state, as seen in our experimental data. This indicates that a model, in which parameters are estimated purely based on mutant flowering times, might miss important aspects of gene expression dynamics. Additional time course data could in the future be obtained at various experimental conditions (temperature, light) as a step towards including the effect of such conditions on the model. A direct advantage is that our parameters have a physical interpretation (e.g. activation strength, cooperativity, etc).

When analysing for which genes the model predictions were of better quality, the effects of an *SVP* overexpression mutant and an *FT* knock-out mutant on flowering time were predicted less accurately compared to other mutants (including *SVP* knock-out and *FT* overexpression mutants). *FT* and *SVP* are connected to each other in the network, which could indicate that in this part of the network the model needs refinement. In particular, given that *SVP* overexpression results in lower *FT* expression, the fact that both *SVP* overexpression and *FT* knock-out were not well predicted indicates that the effect of lower *FT* levels, either directly on *AP1* or more indirect via *SOC1*, is not perfectly captured. It is however also important to consider that the *FT* levels used as input in our model are relatively low, which is related to the fact that they are not measured at the peak of diurnal expression of *FT*. Another aspect to consider is that *FLC* and *SVP* are present as external inputs in the model and are not directly modelled; if a mutation in one of these impacts the other as well, the model would miss such effect, which would deteriorate prediction performance. This might indeed be the case, according to ChIP-seq data [35,36].

Clearly, there are several directions to expand our work. We do not specifically represent protein and RNA separately; currently the state of the art in the proteomics field does not allow high-throughput and precise quantification of protein levels during the vegetative phase of plant development. Recent evidence indicates however that for at least one component in the model, SVP, the effect of protein stability is important [52]. In theory, for the differential effects of *soc1* vs. *agl24* mutation, for which we provide an explanation in terms of a difference in a specific parameter in the model, difference in protein levels in spite of similarity in RNA levels could also be relevant, although there is currently no experimental data that indicates this.

In general, the amount of detail in the model will always be a compromise. This holds as well for the type of interactions in the network. Currently, regulatory interactions are modelled, whereas protein-protein interactions are not explicitly included. Nevertheless, the way in which regulatory inputs are combined gives an implicit representation of the way in which proteins interact with each other. Although the importance of complex formation for the components of the network is clear [49,53], one reason why at our level of detail protein complexes can be excluded might be that they are mainly relevant for specifying the correct regulatory interactions (which are explicitly defined in the model equations) and less so for the kinetics of the model. Depending on the availability of proteomics data, it would however be straightforward to include e.g. protein dimerization explicitly in our equations. Another relevant addition could be to include post-translational modifications such as phosphorylation, which are known to be relevant for some of the components of the flowering time regulatory network [54].

Currently, we focused on a core set of genes involved in integrating various flowering time signals. Given that input from the environment converges on various components of the flowering integration network, an exciting follow-up step will be to incorporate environmental cues as the next layer of information in the gene regulatory network. This could include both direct environmental effects on some of the model components, or modelling complete upstream pathways. As an example of direct environmental influence that could be modelled, recent data indicates that the above mentioned effects of SVP protein stability as well alternative splicing of the flowering time regulator Flowering Locus M (FLM) depend on temperature [55]. To include the former, although protein levels are not explicitly present in our model, an effect of temperature on stability could be represented by changing the *SVP* decay parameter; for FLM, additional equations describing the two isoforms would be needed. As for the modelling of upstream pathways, in a recent overview of known effects of mutations, ~150 genes were listed as being currently known to impact flowering time [56]. It remains to be seen which would be the best approach to include such genes and whether it is essential to include all of them for reliable predictions. Given sufficient time-course data it might be possible to use the same approach as presented here. However, it would also be an option to focus detailed modelling efforts on particular parts of the network; for example, for the influence of light on the circadian clock, models have already been developed [4–6] and these could be connected to our model. Other parts of the network could be treated in a more coarse grained, statistical approach.

To conclude, we present a dynamic and predictive model for flowering time regulation. Our work presents a framework for studying the mechanisms of flowering time regulation, by addressing how different quantitative inputs are combined into a single quantitative output, the timing of flowering.

## Methods

### Plant materials and growth conditions

For the time-course gene expression studies Arabidopsis Col-0 wild type plants were grown under long-day conditions (16 hrs light, 8 hrs dark; 21°C) on rockwool and received 1 g/L Hyponex plant food solution two times per week. Rosette leaves and shoot apical meristem enriched material was harvested daily at ZT3 from seven plants per sample in duplicate.

Plants for flowering time analysis were grown in growth chambers with controlled environment (23°C, 65% relative humidity) under long-day conditions (16 hrs light, 8 hrs dark). Plants were raised on soil under a mixture of Cool White and Gro-Lux Wide Spectrum fluorescent lights, with a fluorescence rate of 125 to 175 mmol m^-2^ s^1^. For flowering time measurements, the total number of primary rosette leaves was scored at visual bolting. The position of the plants from the different genotypes were randomized in the trays, and the flowering time phenotype was recorded without prior knowledge of the genotype. Plants for microarray experiments were grown on soil in growth chambers (23°C, 65% relative humidity) under short-day conditions (8 hrs light, 16 hrs dark) for 25 days (Col-0, *soc1–6* (SALK_138131), *agl24* (SALK_095007), *flc-3*) or 28 days (*fd-3*). Flowering was induced by shifting plants to long-day conditions (16 hrs light, 8 hrs dark).

### Quantitative qRT-PCR data

RNA was isolated from the plant samples (max 100 mg of grinded plant material) using the InviTrap Spin Plant RNA Mini Kit. Subsequently, a DNAse (Invitrogen) treatment was performed, which was stopped with 1μL of a 20 mM EDTA solution and 10 minutes incubation at 65°C. Total RNA concentration was measured, and 1 μg RNA was used to perform cDNA synthesis by the Taqman MultiScribe Reverse Transcriptase kit (LifeTechnologies). qRT-PCR was performed with the SYBR green mix from BioRad using the gene specific oligonucleotides indicated in Table D in S1 File. *YELLOW-LEAF-SPECIFIC GENE8 (YLS8)* was implemented as reference gene for the analyses.

The relative gene expression was given by *E*_*target*_ = 2^Δ*Ct*^, where *Ct* stands for the threshold cycle and Δ*Ct* = *Ct*_*target*_—*Ct*_*reference*_. From that, the absolute abundance was estimated by *A*_*target*_ = *E*_*target*_ × *s*, where *s* stands for a scaling factor obtained by dividing the average abundance that a transcript reaches in a cell by the highest *E*_*target*_ value among all samples, and multiplying by an assumed maximal protein abundance. Since a linear relationship between abundances of RNA and protein is assumed in the model, the average transcript abundance was adjusted based on average abundance of a protein in cell. An available estimate for the range of protein abundance is between 400nM and 1400nM [57]. From this range, the average abundance for the flowering time gene products was arbitrarily chosen (500nM). This means that the maximum absolute expression among all samples is equal to 500nM (Fig. 2). Scaled expression values used in parameter estimation are available in S1 Dataset, which also contains model source code (see below).

### Microarray data

Microarray time series experiments were performed as previously described [58] using RNA isolated from manually dissected shoot apices of Col-0, *soc1–6*, *agl24*, and *fd-3*. Briefly, biotinylated probes were prepared from 1 μg of total RNA using the MessageAmp II-Biotin Enhanced Kit (Ambion) following the manufacturer’s instructions and hybridized to Arabidopsis ATH1–121501 gene expression array (Affymetrix). Arrays were washed on a GeneChip Fluidics Station 450 (affymetrix) and scanned on an Affymetrix GeneChip Scanner 7G using default settings. Expression data for Col-0, *soc-6*, *agl24*, and *fd-3* have been deposited with ArrayExpress (E-MEXP-4001). Expression data for *flc-3* (ArrayExpress: E-MEXP-2041) have previously been published [59]. The probe intensities were normalized and the gene expression estimates were obtained using the gcRMA library of R/Bioconductor [60].

### The model

The regulatory interactions shown in Fig. 1 were modelled by equations based on Hill kinetics. It was assumed that spatial aspects could be ignored (except for FT transport); hence the interactions between the components are described by a set of ordinary differential equations (ODEs). Furthermore, only proteins were explicitly modelled, and a linear relationship between RNA levels and protein levels was assumed. The model is composed of the following equations:

$$\frac{dx_{FT}}{dt}=\beta_{1}\left(\frac{K_{1}}{K_{1}+x_{SVP,l}}\right)\left(\frac{K_{2}}{K_{2}+x_{FLC,l}}\right)-d_{1}x_{FT}$$ (1)

$$\frac{dx_{AGL24}}{dt}=\beta_{2}\left(\frac{x_{SOC1}}{K_{3}+x_{SOC1}}\right)-d_{2}x_{AGL24}$$ (2)

$$\frac{dx_{SOC1}}{dt}=\left[\left(\frac{\beta_{3}x_{AGL24}}{K_{4}+x_{AGL24}}\right)+\left(\frac{\beta_{4}x_{SOC1}}{K_{5}+x_{SOC1}}\right)+\left(\frac{\beta_{5}x_{FT,t-\Delta }}{K_{6}+x_{FT,t-\Delta }}\right)\left(\frac{x_{FD}}{K_{7}+x_{FD}}\right)\right]\times \left(\frac{K_{8}}{K_{8}+x_{SVP,m}}\right)\left(\frac{K_{9}}{K_{9}+x_{FLC,m}}\right)-d_{3}x_{SOC1}$$ (3)

$$\frac{dx_{LFY}}{dt}=\left[\left(\frac{\beta_{6}x_{AGL24}}{K_{10}+x_{AGL24}}\right)+\left(\frac{\beta_{7}x_{SOC1}}{K_{11}+x_{SOC1}}\right)+\left(\frac{\beta_{8}x_{AP1}}{K_{12}+x_{AP1}}\right)\right]-d_{4}x_{LFY}$$ (4)

$$\frac{dx_{AP1}}{dt}=\left[\left(\frac{\beta_{9}x_{LFY}^{n}}{K_{13}^{n}+x_{LFY}^{n}}\right)+\left(\frac{\beta_{10}x_{FT,t-\Delta }}{K_{14}+x_{FT,t-\Delta }}\right)+\left(\frac{\beta_{11}x_{FD}}{K_{15}+x_{FD}}\right)\right]-d_{5}x_{AP1}$$ (5)

$$\frac{dx_{FD}}{dt}=\beta_{12}\left(\frac{x_{LFY}}{K_{16}+x_{LFY}}\right)-d_{6}x_{FD}$$ (6)

For *FLC* and *SVP*, gene expression is represented in the leaves (*x*_*FLC*,*l*_ and *x*_*SVP*,*l*_) and meristem (*x*_*FLC*,*m*_ and *x*_*SVP*,*m*_). For all the other genes, the variables correspond to expression in the meristem. Note that for *SVP* and *FLC* there are no equations; they act as external inputs in the model, and their regulation is not explicitly modelled. The parameters in the equations have the following meaning (see Tables B-C in S1 File for further details): parameters β and K stand for the maximum transcription rate and for the abundance at half-maximum transcription rate, respectively; d_i_ stands for the degradation rate of the products of gene *i*; Δ stands for the time needed for transporting FT from the leaves to the meristem; x_FT,t-Δ_ is the amount of FT in the meristem at time t which is assumed to be equal to that in the leaves at time t-Δ; and *n* is the Hill coefficient describing cooperativity in the regulation of *AP1* by LFY.

Equations (1–6) are based on the following specific assumptions: (I) *SVP* and *FLC* bind to *FT* and *SOC1* promoters as a dimer. This is implicitly represented by the multiplication of the Hill terms associated to the S*VP-* and *FLC-*mediated regulations of *FT* and *SOC1*. (II) FD requires dimerization with FT in order to activate *SOC1* expression. (III) FD can activate *AP1* as a monomer. (IV) Recently it was shown that in rice the interaction between FT and FD is bridged by a 14–3–3 protein [61] and probably this is also the case in Arabidopsis; nevertheless, we did not include 14–3–3s in our model, because these proteins seem to be highly abundant and hence not limiting for floral induction. The specific form of the equations and the assumptions that they represent were adjusted by assessing the fitting and the flowering time predictions of variants for the five equations. In addition, to obtain a good fit for the equation associated to *AP1*, the degree of cooperativity (*n*) for the LFY-mediated regulation of *AP1* was set to *n* = 3. Model source code is available as S1 Dataset.

### Parameter estimation

In the model, the expression dynamics *x*_*i*_ of a gene *i* depends on the parameter values associated to $\frac{dx_{i}}{dt}$ and on the expression values over the time-course of the direct regulators of *i*. To independently fit an equation $\frac{dx_{i}}{dt}$ to its corresponding time-course, the expressions of the direct regulators of *i* in the right-hand side of the equation were taken from the data, and interpolated with a polynomial fit. This decoupling method has previously been described in full detail [14]. By applying this method, it is possible to find the parameters for each equation without knowing the parameters associated to the other equations; thus, alleviating the high computational demand put on the search algorithm by the total number of parameters. This optimization step was carried out by the *MultiStart* solver implemented in MATLAB (R2012a, The MathWorks UK, Cambridge). The parameters were then input in the whole systems of equations as starting point for a second optimization step. In this second step, the equations were solved as a system and the expressions of the direct regulators of *i* were taken from their associated ordinary differential equation solutions. This was carried out by the *lsqnonlin* solver (implemented in MATLAB) to fine-tune the fitting obtained by the first optimization step.

To assess the goodness of fit for each gene, the normalized root mean square error (NRMSE) was used, which equals$\frac{RMSE}{x_{\max}-x_{\min}}$, with RMSE equal to$\sqrt{\frac{\sum_{i}{\left(x_{i}^{\exp}-x_{i}^{pred}\right)}^{2}}{n}}$; here *x*_*max*_, *x*_*min*_ are the maximum and minimum observed expression value; x_i_^exp^ and x_i_^pred^ are the experimental and predicted values at time *i*; and the sum is over all *n* timepoints.

### Model simulations

The equations were solved using MATLAB, integrated with the stiff solver *ode23s*. For simulations of gene expressions of Arabidopsis wild type grown at 23°C/LD, the initial gene abundances were taken equal to the first expression time-points and the parameters were the same as described in Table C in S1 File. To simulate gene expression in mutants, the expression associated to a mutated gene *i* was fixed to a constant value *x*_*i*_ = *k*_*mut*_. For the knock-out null mutants (*ft-10*, *fd-3*, *flc-3*), the values of *k*_*mut*_ were adjusted to zero; and for the knockdown mutants (not null mutations), *k*_*mut*_ values were adjusted to a small percentage of the expression of *i* observed in the first time-point from wild type Col-0 (Table E in S1 File). For the overexpression mutants, the values of *k*_*mut*_ were set to five times the maximum absolute expression among all samples (2500nM).

To assess the model predictions of changes in gene expression we compared predicted relative changes with relative changes obtained with microarray data. To do so, we calculated the predicted total amount of expression (integral of the predicted time-course from day 0 to day 20) using the *trapz* function in MATLAB. Subsequently, these values were scaled by subtracting the wild type value and then dividing by the wild type value. Similarly, the experimental relative change was calculated based on the microarray data. Note that comparing these values focusses on the effect of a mutation on dynamics of genes in the network over the complete time-course and as such takes into account the fact that the experimental conditions of the microarray experiment cannot directly be simulated (flowering-inducing shift from short-day to long-day conditions).

### Model predictions of flowering time

The predictions of flowering time were based on *AP1* expression. For that it was assumed that, at a molecular level, *Arabidopsis* undergoes the floral transition in the moment that *AP1* expression initiates. Therefore, according to our experimental *AP1* time series, for wild type Col-0, the floral transition takes place between days 12 and 13 after germination. For simplification, we take the exact day 12.6 because it corresponds to the average number of rosette leaves (RLs) observed at the onset of flowering for wild type Col-0. To estimate the flowering time from mutant simulations, we use the time in which *AP1* expression reaches the same simulated expression value as obtained at day 12.6 for wild type Col-0. This implies that the *AP1* expression threshold for triggering the floral transition is the same for different plant growth conditions and mutants. Because flowering times are usually reported in number of rosette leaves (RLs) we subsequently scaled the predicted days to RLs by assuming a linear relationship between the number of RLs observed at the onset of flowering and the time in days after germination that *Arabidopsis thaliana* undergoes the floral transition at a molecular level.

In addition to the set of mutants obtained in consistent conditions in this work, we also included existing mutant data. Wild type Col-0 flowering time in these experiments is somewhat different from that observed in our experiments. In addition, flowering times in literature are mostly reported in rosette leaves (RL), and not directly in days. To be able to integrate these data, we scaled existing mutant data with a linear factor which is chosen in such a way as to scale the wild type Col-0 flowering time to 12.6 RL.

## Supporting Information

S1 File Supplementary Figures and Tables. (DOCX)

S1 Dataset Model source code and time-course expression data used for parameter estimation. Contains model source code (MATLAB) and RT-PCR data used for parameter estimation. Main model file is “ode_application_final.m”. This uses two files (LoadParametersFromFile_Leaf.m and LoadParametersFromFile_Meristem.m) to read the parameter values; two files that contain the RT-PCR data (dataset_qPCR_normalized_Leaf.m and dataset_qPCR_normalized_Meristem.m) and two files with ODEs (ode_equation_FT_leaf.m and ode_equations.m).
(ZIP)

## References

[1] Srikanth A, Schmid M (2011) Regulation of flowering time: all roads lead to Rome. Cell Mol Life Sci 68: 2013–2037. 10.1007/s00018-011-0673-y 21611891 PMC11115107

[2] Andres F, Coupland G (2012) The genetic basis of flowering responses to seasonal cues. Nat Rev Genet 13: 627–639. 10.1038/nrg3291 22898651

[3] Chew YH, Smith RW, Jones HJ, Seaton DD, Grima R, et al (2014) Mathematical models light up plant signaling. Plant Cell 26: 5–20. 10.1105/tpc.113.120006 24481073 PMC3963593

[4] Gould PD, Ugarte N, Domijan M, Costa M, Foreman J, et al (2013) Network balance via CRY signalling controls the Arabidopsis circadian clock over ambient temperatures. Mol Syst Biol 9: 650 10.1038/msb.2013.7 23511208 PMC3619941

[5] Keily J, Macgregor DR, Smith RW, Millar AJ, Halliday KJ, et al (2013) Model selection reveals control of cold signalling by evening-phased components of the plant circadian clock. Plant J. 10.1111/tpj.12303 23909712 PMC4278413

[6] Pokhilko A, Fernandez AP, Edwards KD, Southern MM, Halliday KJ, et al (2012) The clock gene circuit in Arabidopsis includes a repressilator with additional feedback loops. Mol Syst Biol 8: 574 10.1038/msb.2012.6 22395476 PMC3321525

[7] Schmal C, Reimann P, Staiger D (2013) A Circadian Clock-Regulated Toggle Switch Explains AtGRP7 and AtGRP8 Oscillations in Arabidopsis thaliana. PLoS Comput Biol 9. 10.1371/journal.pcbi.1002986 PMC3610657 23555221

[8] Jonsson H, Heisler MG, Shapiro BE, Meyerowitz EM, Mjolsness E (2006) An auxin-driven polarized transport model for phyllotaxis. Proc Natl Acad Sci U S A 103: 1633–1638. 16415160 10.1073/pnas.0509839103 PMC1326488

[9] van Mourik S, Kaufmann K, van Dijk AD, Angenent GC, Merks RM, et al (2012) Simulation of organ patterning on the floral meristem using a polar auxin transport model. PLoS One 7: e28762 10.1371/journal.pone.0028762 22291882 PMC3264561

[10] Peret B, Middleton AM, French AP, Larrieu A, Bishopp A, et al (2013) Sequential induction of auxin efflux and influx carriers regulates lateral root emergence. Mol Syst Biol 9: 699 10.1038/msb.2013.43 24150423 PMC3817398

[11] Grieneisen VA, Xu J, Maree AF, Hogeweg P, Scheres B (2007) Auxin transport is sufficient to generate a maximum and gradient guiding root growth. Nature 449: 1008–1013. 17960234 10.1038/nature06215

[12] Salazar JD, Saithong T, Brown PE, Foreman J, Locke JC, et al (2009) Prediction of photoperiodic regulators from quantitative gene circuit models. Cell 139: 1170–1179. 10.1016/j.cell.2009.11.029 20005809

[13] Song YH, Smith RW, To BJ, Millar AJ, Imaizumi T (2012) FKF1 conveys timing information for CONSTANS stabilization in photoperiodic flowering. Science 336: 1045–1049. 10.1126/science.1219644 22628657 PMC3737243

[14] van Mourik S, van Dijk AD, de Gee M, Immink RG, Kaufmann K, et al (2010) Continuous-time modeling of cell fate determination in Arabidopsis flowers. BMC Syst Biol 4: 101 10.1186/1752-0509-4-101 20649974 PMC2922098

[15] Espinosa-Soto C, Padilla-Longoria P, Alvarez-Buylla ER (2004) A gene regulatory network model for cell-fate determination during Arabidopsis thaliana flower development that is robust and recovers experimental gene expression profiles. Plant Cell 16: 2923–2939. 15486106 10.1105/tpc.104.021725 PMC527189

[16] Mendoza L, Alvarez-Buylla ER (1998) Dynamics of the genetic regulatory network for Arabidopsis thaliana flower morphogenesis. J Theor Biol 193: 307–319. 9714934 10.1006/jtbi.1998.0701

[17] Sanchez-Corrales YE, Alvarez-Buylla ER, Mendoza L (2010) The Arabidopsis thaliana flower organ specification gene regulatory network determines a robust differentiation process. J Theor Biol 264: 971–983. 10.1016/j.jtbi.2010.03.006 20303988

[18] Jung C, Muller AE (2009) Flowering time control and applications in plant breeding. Trends Plant Sci 14: 563–573. 10.1016/j.tplants.2009.07.005 19716745

[19] Dong Z, Danilevskaya O, Abadie T, Messina C, Coles N, et al (2012) A gene regulatory network model for floral transition of the shoot apex in maize and its dynamic modeling. PLoS One 7: e43450 10.1371/journal.pone.0043450 22912876 PMC3422250

[20] Jaeger KE, Pullen N, Lamzin S, Morris RJ, Wigge PA (2013) Interlocking feedback loops govern the dynamic behavior of the floral transition in Arabidopsis. Plant Cell 25: 820–833. 10.1105/tpc.113.109355 23543784 PMC3634691

[21] Satake A, Kawagoe T, Saburi Y, Chiba Y, Sakurai G, et al (2013) Forecasting flowering phenology under climate warming by modelling the regulatory dynamics of flowering-time genes. Nat Commun 4: 2303 10.1038/ncomms3303 23941973

[22] Welch SM, Roe JL, Dong ZS (2003) A genetic neural network model of flowering time control in Arabidopsis thaliana. Agronomy Journal 95: 71–81.

[23] Pose D, Yant L, Schmid M (2012) The end of innocence: flowering networks explode in complexity. Curr Opin Plant Biol 15: 45–50. 10.1016/j.pbi.2011.09.002 21974961

[24] Jang S, Torti S, Coupland G (2009) Genetic and spatial interactions between FT, TSF and SVP during the early stages of floral induction in Arabidopsis. Plant J 60: 614–625. 10.1111/j.1365-313X.2009.03986.x 19656342

[25] Helliwell CA, Wood CC, Robertson M, James Peacock W, Dennis ES (2006) The Arabidopsis FLC protein interacts directly in vivo with SOC1 and FT chromatin and is part of a high-molecular-weight protein complex. Plant J 46: 183–192. 16623882 10.1111/j.1365-313X.2006.02686.x

[26] Li D, Liu C, Shen L, Wu Y, Chen H, et al (2008) A repressor complex governs the integration of flowering signals in Arabidopsis. Dev Cell 15: 110–120. 10.1016/j.devcel.2008.05.002 18606145

[27] Tao Z, Shen L, Liu C, Liu L, Yan Y, et al (2012) Genome-wide identification of SOC1 and SVP targets during the floral transition in Arabidopsis. Plant J 70: 549–561. 10.1111/j.1365-313X.2012.04919.x 22268548

[28] Mathieu J, Warthmann N, Kuttner F, Schmid M (2007) Export of FT protein from phloem companion cells is sufficient for floral induction in Arabidopsis. Curr Biol 17: 1055–1060. 17540570 10.1016/j.cub.2007.05.009

[29] Corbesier L, Vincent C, Jang S, Fornara F, Fan Q, et al (2007) FT protein movement contributes to long-distance signaling in floral induction of Arabidopsis. Science 316: 1030–1033. 17446353 10.1126/science.1141752

[30] Abe M, Kobayashi Y, Yamamoto S, Daimon Y, Yamaguchi A, et al (2005) FD, a bZIP protein mediating signals from the floral pathway integrator FT at the shoot apex. Science 309: 1052–1056. 16099979 10.1126/science.1115983

[31] Wigge PA (2013) Ambient temperature signalling in plants. Curr Opin Plant Biol. 10.1016/j.pbi.2013.08.004 24021869

[32] Yoo SK, Chung KS, Kim J, Lee JH, Hong SM, et al (2005) CONSTANS activates SUPPRESSOR OF OVEREXPRESSION OF CONSTANS 1 through FLOWERING LOCUS T to promote flowering in Arabidopsis. Plant Physiol 139: 770–778. 16183837 10.1104/pp.105.066928 PMC1255994

[33] Wigge PA, Kim MC, Jaeger KE, Busch W, Schmid M, et al (2005) Integration of spatial and temporal information during floral induction in Arabidopsis. Science 309: 1056–1059. 16099980 10.1126/science.1114358

[34] Hartmann U, Hohmann S, Nettesheim K, Wisman E, Saedler H, et al (2000) Molecular cloning of SVP: a negative regulator of the floral transition in Arabidopsis. Plant J 21: 351–360. 10758486 10.1046/j.1365-313x.2000.00682.x

[35] Gregis V, Andres F, Sessa A, Guerra RF, Simonini S, et al (2013) Identification of pathways directly regulated by SHORT VEGETATIVE PHASE during vegetative and reproductive development in Arabidopsis. Genome Biol 14: R56 10.1186/gb-2013-14-6-r56 23759218 PMC3706845

[36] Deng W, Ying H, Helliwell CA, Taylor JM, Peacock WJ, et al (2011) FLOWERING LOCUS C (FLC) regulates development pathways throughout the life cycle of Arabidopsis. Proc Natl Acad Sci U S A 108: 6680–6685. 10.1073/pnas.1103175108 21464308 PMC3081018

[37] Immink RG, Pose D, Ferrario S, Ott F, Kaufmann K, et al (2012) Characterization of SOC1’s central role in flowering by the identification of its upstream and downstream regulators. Plant Physiol 160: 433–449. 10.1104/pp.112.202614 22791302 PMC3440217

[38] Michaels SD, Ditta G, Gustafson-Brown C, Pelaz S, Yanofsky M, et al (2003) AGL24 acts as a promoter of flowering in Arabidopsis and is positively regulated by vernalization. Plant J 33: 867–874. 12609028 10.1046/j.1365-313x.2003.01671.x

[39] Lee J, Oh M, Park H, Lee I (2008) SOC1 translocated to the nucleus by interaction with AGL24 directly regulates leafy. Plant J 55: 832–843. 10.1111/j.1365-313X.2008.03552.x 18466303

[40] Wagner D, Sablowski RW, Meyerowitz EM (1999) Transcriptional activation of APETALA1 by LEAFY. Science 285: 582–584. 10417387 10.1126/science.285.5427.582

[41] Kaufmann K, Wellmer F, Muino JM, Ferrier T, Wuest SE, et al (2010) Orchestration of floral initiation by APETALA1. Science 328: 85–89. 10.1126/science.1185244 20360106

[42] Hempel FD, Weigel D, Mandel MA, Ditta G, Zambryski PC, et al (1997) Floral determination and expression of floral regulatory genes in Arabidopsis. Development 124: 3845–3853. 9367440 10.1242/dev.124.19.3845

[43] Hill AV (1910) The possible effects of the aggregation of the molecules of haemoglobin on its dissociation curves. J Physiol 40.

[44] Giakountis A, Coupland G (2008) Phloem transport of flowering signals. Curr Opin Plant Biol 11: 687–694. 10.1016/j.pbi.2008.10.003 18977685

[45] Torti S, Fornara F, Vincent C, Andres F, Nordstrom K, et al (2012) Analysis of the Arabidopsis shoot meristem transcriptome during floral transition identifies distinct regulatory patterns and a leucine-rich repeat protein that promotes flowering. Plant Cell 24: 444–462. 10.1105/tpc.111.092791 22319055 PMC3315226

[46] Michaels SD, Amasino RM (1999) FLOWERING LOCUS C encodes a novel MADS domain protein that acts as a repressor of flowering. Plant Cell 11: 949–956. 10330478 10.1105/tpc.11.5.949 PMC144226

[47] van Leeuwen W, Ruttink T, Borst-Vrenssen AW, van der Plas LH, van der Krol AR (2001) Characterization of position-induced spatial and temporal regulation of transgene promoter activity in plants. J Exp Bot 52: 949–959. 11432912 10.1093/jexbot/52.358.949

[48] Hames C, Ptchelkine D, Grimm C, Thevenon E, Moyroud E, et al (2008) Structural basis for LEAFY floral switch function and similarity with helix-turn-helix proteins. EMBO J 27: 2628–2637. 10.1038/emboj.2008.184 18784751 PMC2567413

[49] de Folter S, Immink RG, Kieffer M, Parenicova L, Henz SR, et al (2005) Comprehensive interaction map of the Arabidopsis MADS Box transcription factors. Plant Cell 17: 1424–1433. 15805477 10.1105/tpc.105.031831 PMC1091765

[50] Pajoro A, Biewers S, Dougali E, Leal Valentim F, Mendes MA, et al (2014) The (r)evolution of gene regulatory networks controlling Arabidopsis plant reproduction: a two-decade history. J Exp Bot 65: 4731–4745. 10.1093/jxb/eru233 24913630

[51] Wilczek AM, Roe JL, Knapp MC, Cooper MD, Lopez-Gallego C, et al (2009) Effects of genetic perturbation on seasonal life history plasticity. Science 323: 930–934. 10.1126/science.1165826 19150810

[52] Lee JH, Ryu HS, Chung KS, Pose D, Kim S, et al (2013) Regulation of temperature-responsive flowering by MADS-box transcription factor repressors. Science 342: 628–632. 10.1126/science.1241097 24030492

[53] Immink RG, Tonaco IA, de Folter S, Shchennikova A, van Dijk AD, et al (2009) SEPALLATA3: the ‘glue’ for MADS box transcription factor complex formation. Genome Biol 10: R24 10.1186/gb-2009-10-2-r24 19243611 PMC2688274

[54] Wang Y, Liu C, Yang D, Yu H, Liou YC (2010) Pin1At encoding a peptidyl-prolyl cis/trans isomerase regulates flowering time in Arabidopsis. Mol Cell 37: 112–122. 10.1016/j.molcel.2009.12.020 20129060

[55] Pose D, Verhage L, Ott F, Yant L, Mathieu J, et al. (2013) Temperature-dependent regulation of flowering by antagonistic FLM variants. Nature. 10.1038/nature12633 24067612

[56] Lloyd J, Meinke D (2012) A comprehensive dataset of genes with a loss-of-function mutant phenotype in Arabidopsis. Plant Physiol 158: 1115–1129. 10.1104/pp.111.192393 22247268 PMC3291275

[57] Ghaemmaghami S, Huh WK, Bower K, Howson RW, Belle A, et al (2003) Global analysis of protein expression in yeast. Nature 425: 737–741. 14562106 10.1038/nature02046

[58] Schmid M, Uhlenhaut NH, Godard F, Demar M, Bressan R, et al (2003) Dissection of floral induction pathways using global expression analysis. Development 130: 6001–6012. 14573523 10.1242/dev.00842

[59] Mathieu J, Yant LJ, Murdter F, Kuttner F, Schmid M (2009) Repression of flowering by the miR172 target SMZ. PLoS Biol 7: e1000148 10.1371/journal.pbio.1000148 19582143 PMC2701598

[60] Gentleman RC, Carey VJ, Bates DM, Bolstad B, Dettling M, et al (2004) Bioconductor: open software development for computational biology and bioinformatics. Genome Biol 5: R80 15461798 10.1186/gb-2004-5-10-r80 PMC545600

[61] Taoka K, Ohki I, Tsuji H, Furuita K, Hayashi K, et al (2011) 14–3–3 proteins act as intracellular receptors for rice Hd3a florigen. Nature 476: 332–335. 10.1038/nature10272 21804566

