# Supplementary material for: A Quantitative and Dynamic Model of the Arabidopsis Flowering Time Gene Regulatory Network
Source: PLoS One. 2015 Feb 26;10(2):e0116973. doi: 10.1371/journal.pone.0116973 (PMC4342252; doi:10.1371/journal.pone.0116973)
Supplement: S1 File — (DOCX) [file pone.0116973.s001.docx]

**A quantitative and dynamic model of the Arabidopsis flowering time gene regulatory network**

F. Leal Valentim^1^, S. van Mourik^2,6^, D. Posé^3,$^, M.C. Kim^3,§^, M. Schmid^3^, R.C.H.J. van Ham^4^, M. Busscher^1^, G.F. Sanchez-Perez^1,7^, J. Molenaar^2^, G.C. Angenent^1,5^, R.G.H. Immink^1^, A. D. J. van Dijk^1,2,6^*

^1^ Bioscience, Plant Research International, Bioscience, Wageningen, The Netherlands.

^2^ Biometris, Wageningen UR, The Netherlands

^3^ Max Planck Institute for Developmental Biology, Molecular Biology, Tübingen, Germany

^4^ Keygene N.V., Wageningen, The Netherlands

^5^ Laboratory of Molecular Biology, Wageningen University, Wageningen, The Netherlands

^6^ Netherlands Consortium for Systems Biology, Amsterdam

^7^ Chair group Bioinformatics, Wageningen University, Wageningen, The Netherlands

^$^ Current Address: Instituto de Hortofruticultura Subtropical y Mediterránea, Universidad de Málaga–Consejo Superior de Investigaciones Científicas, Departamento de Biología Molecular y Bioquímica, Facultad de Ciencias, Universidad de Málaga, 29071 Málaga,

Spain

^§^ Current Address: Division of Applied Life Science (BK21 Plus), Gyeongsang National University, Jinju, Korea

* Corresponding author: aaltjan.vandijk@wur.nl

**SUPPLEMENTARY INFORMATION**

Supplemental Figure A. Flowering time for Arabidopsis mutants.

Supplemental Figure B. Time-course expression of *FLC* and *SVP* in Arabidopsis wild-type.

Supplemental Figure C. Time-course expression of *AP1*, *LFY*, *SOC1* and *AGL24* in Arabidopsis wild-type (WT) and four mutant backgrounds (*soc1*, *agl24*, *fd* and *flc*).

Supplemental Figure D. Comparison of experimental and simulated changes in gene expression for *AP1*, *LFY*, *SOC1* and *AGL24* in mutant backgrounds (*soc1*, a*gl24*, *fd* and *flc*).

Supplemental Figure E. Fit of AP1 equation for various values of the Hill coefficient.

Supplemental Table A. Regulatory relationships among the flowering time transcription factors.

Supplemental Table B. Lower and Upper Bounds for the Model Parameters.

Supplemental Table C. Estimated Model Parameters.

Supplemental Table D. Sequences of oligonucleotides used in qRT-PCR experiments.

Supplemental Table E. Information used to set up the simulations for the knockdown mutants.


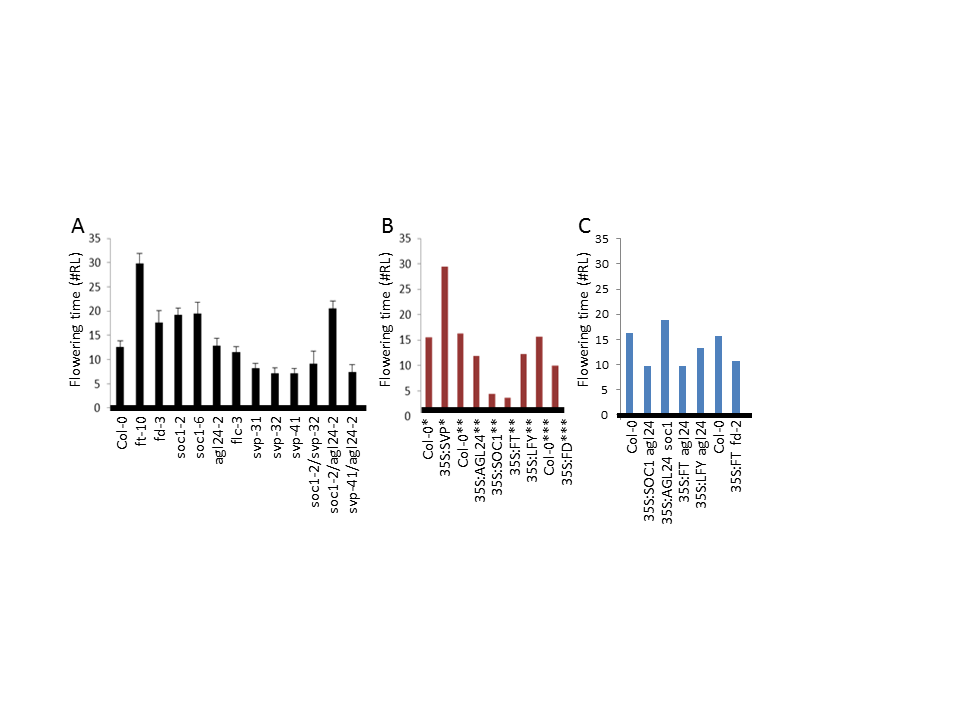


**Supplemental Figure A.** Flowering time for Arabidopsis mutants. (A) Flowering time of knock-down/knock-out mutants measured in this work. Plants were grown in long-day conditions at 23°C. The standard deviation is indicated. (B) Flowering time for transgenic gene overexpression lines obtained from literature survey. Datasources: * [[1](#_ENREF_1)]; ** [[2](#_ENREF_2)]; and ***[[3](#_ENREF_3)]**.** (C) Flowering time for mutants used in model validation**;** obtained from [[1](#_ENREF_1)] except for 35S:FT fd-2 [[3](#_ENREF_3)]. Although similar growth conditions are reported, the flowering time for wild type Col-0 varies among experiments. For this reason, when comparing model predictions with these data, we scaled the literature data based on the ratio between Col-0 flowering time observed in these experiments (panel B, C), and that observed in our experiment (panel A).

**
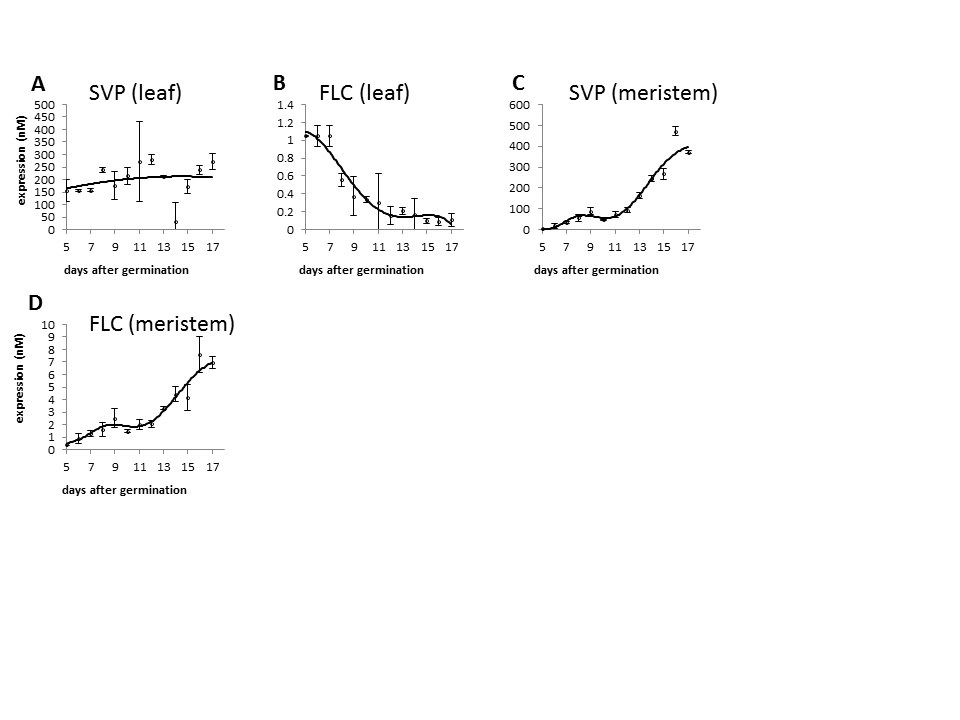
**

**Supplemental Figure B. Time-course expression of *FLC* and *SVP* in Arabidopsis wild-type (WT).** Gene expression was measured by qRT-PCR (shown as dots) of wild type Col-0 plants grown under long-day conditions at 23°C (average and standard deviation are shown). Note that *FLC* and *SVP* are not regulated by other components of the network and hence are present as input factors, and their expression level is not simulated by the model. The continuous lines show interpolated gene expression which was used as the external input. qRT-PCR data indicated as meristem was obtained from meristem enriched material**.**

**
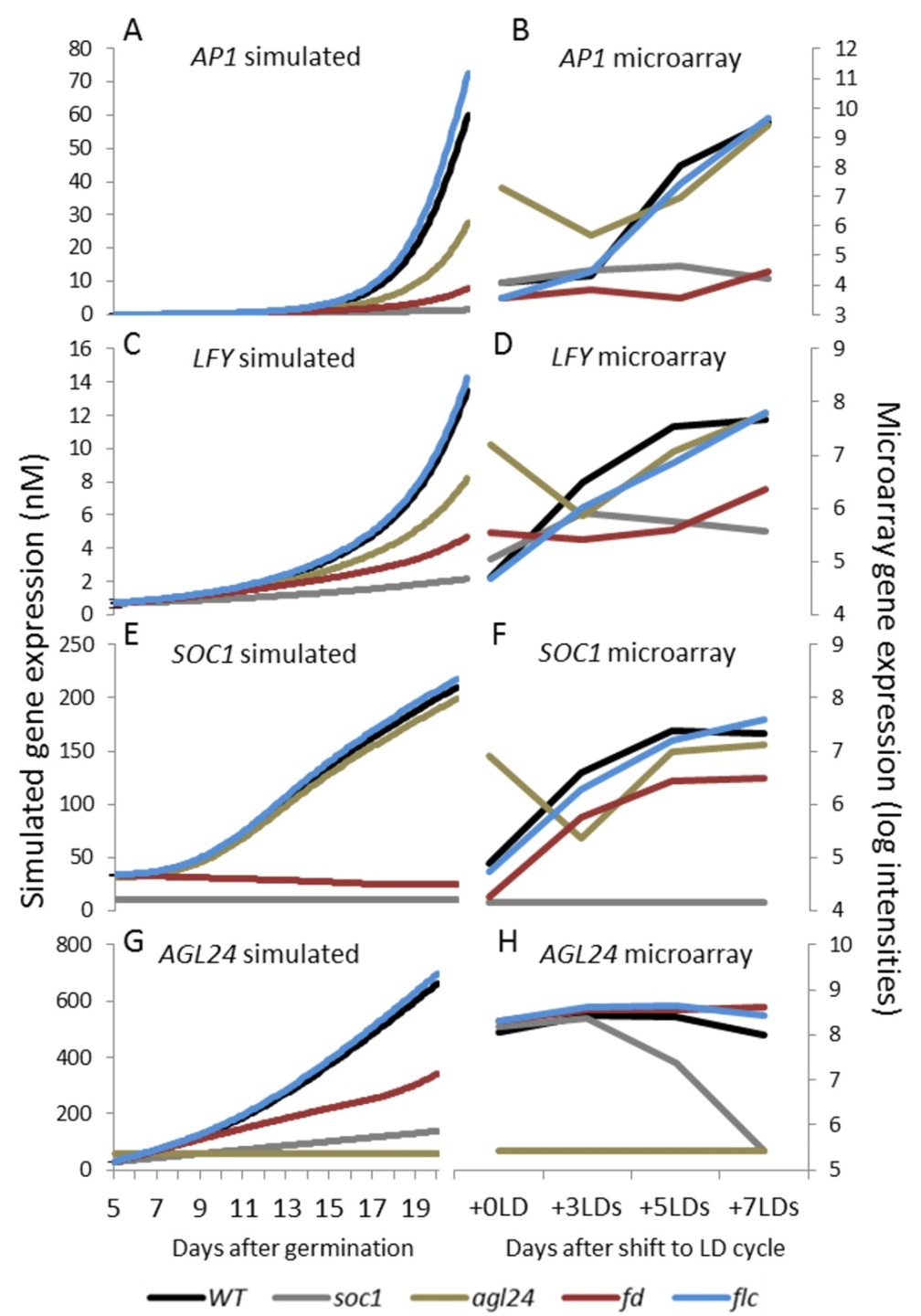
**

Supplemental Figure C. Expression of *AP1*, *LFY*, *SOC1* and *AGL24* in *Arabidopsis* wild-type (WT) and four mutant backgrounds (*soc1*, *agl24*, *fd* and *flc*). Gene expression obtained either from simulations (A,C,E,G) or microarray experiments (B,D,F,H). The simulations show the time-course over 20 days after germination, whilst the microarray data over four time-points after the flowering-inducing shift of plants grown in short-days conditions transferred to long-day conditions (time-points 0, 3, 5 and 7 days after the shift).

**
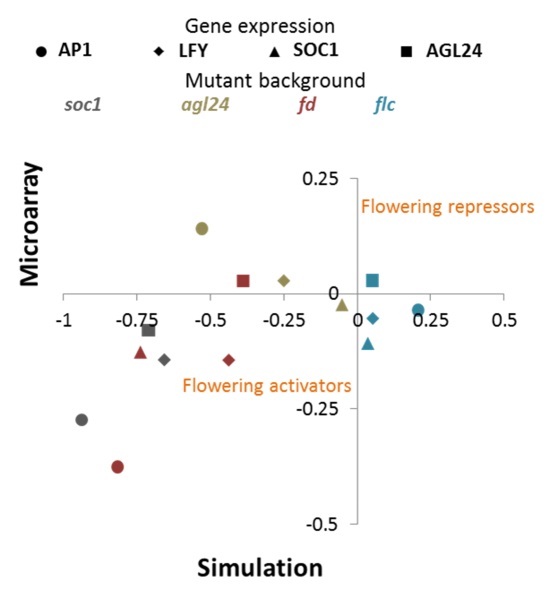
**

Supplemental Figure D. Comparison of experimental and simulated changes in gene expressions for *AP1, LFY, SOC1* and *AGL24* in four mutant backgrounds (*soc1, agl24, fd* and *flc*). The difference in total gene expression between wild type (WT) and mutants was calculated using simulations (*x*-axis) and microarray data (*y*-axis). For each gene, the difference between its expression in WT and in mutant is given by the difference between the area under the WT time-course and that of mutants (SI Fig C); then normalized against the area under the WT time-course. Each colour represents the comparison of one of the four mutants against WT; and each of the compared genes is represented by dots in different shapes. Positive values are obtained when an increase in expression is observed for a mutant compared to that in WT. Pearson's correlation coefficient between simulated and microarray values is 0.67.

**
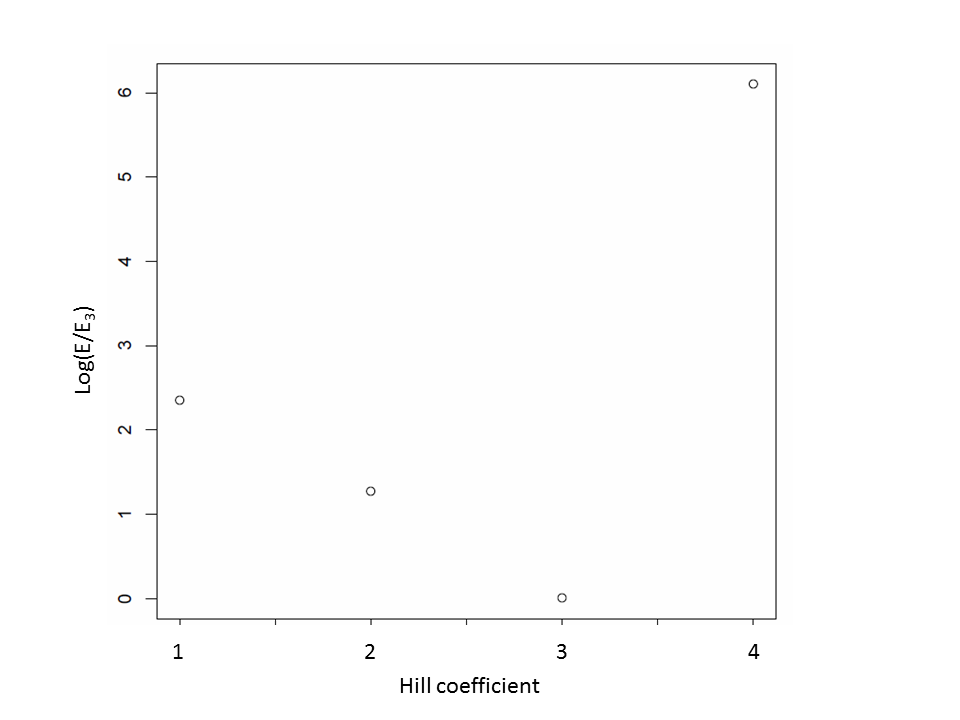
**

**Supplemental Figure E. Fit of AP1 equation for various values of the Hill coefficient.** Hill coefficient n was set to 1,2,3, or 4, and Error Function (E, sum of squared residuals) of resulting fit was divided by the Error Function obtained for n=3 (E_3_). Value shown is log(E/E_3_). Lower value indicates better fit.

Supplemental Table A. Regulatory relationships among the flowering time transcription factors.^a^

| Gene | Activated by | Repressed by |  |
| --- | --- | --- | --- |
| SVP | NA | NA |  |
| FLC | NA | NA |  |
| AGL24 | SOC1 [[4](#_ENREF_4)] | NA |  |
| SOC1 | FT [[5](#_ENREF_5)], SOC1 [[6](#_ENREF_6)], AGL24 [[7](#_ENREF_7)] and FD [[8](#_ENREF_8)] | SVP [[9](#_ENREF_9),[10](#_ENREF_10)], FLC [[11](#_ENREF_11)] |  |
| LFY | AGL24 [[12](#_ENREF_12)], SOC1 [[13](#_ENREF_13)] and AP1 [[14](#_ENREF_14)] | NA |  |
| FT | NA | SVP [[15](#_ENREF_15)] and FLC [[16](#_ENREF_16)] |  |
| FD | LFY [[17](#_ENREF_17)] | NA |  |
| AP1 | LFY [[18](#_ENREF_18)], FT and FD [[3](#_ENREF_3),[8](#_ENREF_8)] | NA |  |

^a^ Note that only regulatory interactions that are relevant before or at the floral transition are included. For example, AP1 regulates additional components of the network after its expression has been initiated, but these interactions are not relevant for the timing of the initial up-regulation of AP1.

Supplemental Table B. Description of the lower and upper bounds for the model parameters.

| Parameter | Description | Principal determinants | Unit | Lower limit | Upper limit | Ref |
| --- | --- | --- | --- | --- | --- | --- |
| *β* | Maximum transcription rate | transcriptional efficiency | $nM*{min}^{-1}$ | $0.001$ | $200$ | a |
| *K* | Abundance at half-maximum transcription rate | binding interface of transcription factor/DNA | $nM$ | $0.001$ | $2000$ | NA |
| *d* | Degradation rate of gene products | protein and RNA stability, pos-transcriptional/-translational regulation | $\min^{-1}$ | $0.001$ | $1$ | b |
| *Δ* | Time needed for transporting FT from the leaves to the meristem |  | days | 0 | 1 | c |

^a^ Based on data in [[19](#_ENREF_19)], we take [0.001, 200] *nM×*min^-1^ as a reasonable range for the possible limit values of ß.

^b^ A range for decay [10^-3^, 10^-1^] min^-1^ is given in [[20](#_ENREF_20)]. We take [10^-3^, 1] min^-1^ as a reasonable range for the possible limit values of decay.

^c^ The range for the delay parameter is adjusted based on the assumption that FT reaches the meristem within at maximum 1 day after being translated in the leaves.

Supplemental Table C. Model parameters estimated from experimental expression time-course data.

| Parameters | Regulatory interaction / gene | Value | Unit |
| --- | --- | --- | --- |
| *K_1_* | SVP 🡪 FT | 0.63 | $nM$ |
| *K_2_* | FLC 🡪 FT | 985 | $nM$ |
| *K_3_* | SOC1 🡪 AGL24 | 125 | $nM$ |
| *K_4_* | AGL24 🡪 SOC1 | 1182 | $nM$ |
| *K_5_* | SOC1 🡪 SOC1 | 695 | $nM$ |
| *K_6_* | FT 🡪 SOC1 | 4.8 | $nM$ |
| *K_7_* | FD 🡪 SOC1 | 2.4 | $nM$ |
| *K_8_* | SVP 🡪 SOC1 | 909 | $nM$ |
| *K_9_* | FLC 🡪 SOC1 | 501 | $nM$ |
| *K_10_* | AGL24 🡪 LFY | 1011 | $nM$ |
| *K_11_* | SOC1 🡪 LFY | 842 | $nM$ |
| *K_12_* | AP1 🡪 LFY | 346 | $nM$ |
| *K_13_* | LFY 🡪 AP1 | 9.82 | $nM$ |
| *K_14_* | FT 🡪 AP1 | 10.1 | $nM$ |
| *K_15_* | FD 🡪 AP1 | 700 | $nM$ |
| *K_16_* | LFY🡪 FD | 7.9 | $nM$ |
| $\boldsymbol{\beta}_{\boldsymbol{1}}$ | SVP/FLC 🡪 FT | 51 | $nM*{min}^{-1}$ |
| $\boldsymbol{\beta}_{\boldsymbol{2}}$ | SOC1🡪 AGL24 | 100 | $nM*{min}^{-1}$ |
| $\boldsymbol{\beta}_{\boldsymbol{3}}$ | AGL24 🡪 SOC1 | 0.52 | $nM*{min}^{-1}$ |
| $\boldsymbol{\beta}_{\boldsymbol{4}}$ | SOC1 🡪 SOC1 | 64 | $nM*{min}^{-1}$ |
| $\boldsymbol{\beta}_{\boldsymbol{5}}$ | FT/FD 🡪 SOC1 | 189 | $nM*{min}^{-1}$ |
| $\boldsymbol{\beta}_{\boldsymbol{6}}$ | AGL24 🡪 LFY | 0.79 | $nM*{min}^{-1}$ |
| $\boldsymbol{\beta}_{\boldsymbol{7}}$ | SOC1 🡪 LFY | 2.4 | $nM*{min}^{-1}$ |
| $\boldsymbol{\beta}_{\boldsymbol{8}}$ | AP1 🡪 LFY | 22 | $nM*{min}^{-1}$ |
| $\boldsymbol{\beta}_{\boldsymbol{9}}$ | LFY 🡪 AP1 | 99.8 | $nM*{min}^{-1}$ |
| $\boldsymbol{\beta}_{\boldsymbol{10}}$ | FT 🡪 AP1 | 10 | $nM*{min}^{-1}$ |
| $\boldsymbol{\beta}_{\boldsymbol{11}}$ | FD🡪 AP1 | 5.0 | $nM*{min}^{-1}$ |
| $\boldsymbol{\beta}_{\boldsymbol{12}}$ | LFY🡪 FD | 8.5 | $nM*{min}^{-1}$ |
| d_1_ | FT | 0.10 | ${min}^{-1}$ |
| d_2_ | AGL24 | 0.0010 | ${min}^{-1}$ |
| d_3_ | SOC1 | 0.11 | ${min}^{-1}$ |
| d_4_ | LFY | 0.017 | ${min}^{-1}$ |
| d_5_ | AP1 | 0.86 | ${min}^{-1}$ |
| d_6_ | FD | 0.0075 | ${min}^{-1}$ |
| *Δ* | FT | 0.50 | $\mathrm{days}$ |
| *n* | LFY🡪 AP1 | 3 | - |

**Supplemental Table D.** Oligonucleotide sequences of oligonucleotides used in qRT-PCR experiments.

| Gene | AtG number | Sequence Forward oligonucleotide | Sequence Reverse oligonucleotide |
| --- | --- | --- | --- |
| *SVP* | At2G22540.1 | PDS3106  5’- GAAGAGAACGAGCG  ACTTGG-3’ | PDS3107  5’- GAGCTCTCGGAGTC  AACAGG-3’ |
| *FLC* | At5G10140.1 | PDS3110  5’- CGAACTCATGTTGA  AGCTTGTT-3’ | PDS3111  5’- GGAGAGTCACCGGA  AGATTG-3’ |
| *AGL24* | At4G24540.1 | PDS3108  5’- CGGAATTGGTGGAT  GAGAAT-3’ | PDS3109  5’- CAGGGAAGTGTCGG  AGTCAT-3’ |
| *SOC1* | At2G45660.1 | PDS3102  5’- AGCTGCAGAAAACG  AGAAGC-3’ | PDS3103  5’- TGAAGAACAAGGTA  ACCCAATG-3’ |
| *LFY* | At5G61850.1 | PDS4778  5’- ATTGGTTCAAGCAC  CACCTC-3’ | PDS4779  5’- ACGGACCGAATAGT  CCCTCT-3’ |
| *FT* | At1G65480.1 | PDS4706  5’- CTGGAACAACCTTT  GGCAAT-3’ | PDS4707  5’- AGCCACTCTCCCTC  TGACAA-3’ |
| *FD* | At4G35900.1 | PDS4758  5’- CACCTCCTGCAACT  GTTCTG-3’ | PDS4759  5’- AGCCTCGAAAGAGG  TGTTGA-3’ |
| *AP1* | At1G69120.1 | PDS3074  5’- TAGGGCTCAACAGG  AGCAGT-3’ | PDS3075  5’- CAGCCAAGGTTGCA  GTTGTA-3’ |
| Ref. gene  *YLS8* | At5G08290.1 | PDS4009  5’-TTACTGTTTCGGTT  GTTCTCCATTT- 3’ | PDS4010  5’- CACTGAATCATGTT  CGAAGCAAGT-3’ |

**Supplemental Table E.** Information used to set up the model simulations for the knockdown mutants.

|  | ***k_mut_* ^a^** | **Reference** |
| --- | --- | --- |
| *soc1-2* | 0.3 | [[4](#_ENREF_4)] |
| *soc1-6* | 0.25 | [[4](#_ENREF_4)] |
| *agl24-2* | 2.0 | [[7](#_ENREF_7)] |
| *agl24-1* | 1.0 | [[4](#_ENREF_4)] |
| *svp-31* | 0.067 | [[9](#_ENREF_9)] |
| *svp-32* | 0.033 | [[9](#_ENREF_9)] |
| *svp-41* | 0.025 | [[9](#_ENREF_9)] |

^a^ To simulate gene expression in mutants, the expression associated to a knockdown mutant was set to $k_{mut}$; the value of *k_mut_* was adjusted to a fraction of the expression of $i$ observed in the first time-point from wild type Col-0.

**References**

1. Yu H, Xu Y, Tan EL, Kumar PP (2002) AGAMOUS-LIKE 24, a dosage-dependent mediator of the flowering signals. Proc Natl Acad Sci U S A 99: 16336-16341.

2. Lee JH, Yoo SJ, Park SH, Hwang I, Lee JS, et al. (2007) Role of SVP in the control of flowering time by ambient temperature in Arabidopsis. Genes Dev 21: 397-402.

3. Wigge PA, Kim MC, Jaeger KE, Busch W, Schmid M, et al. (2005) Integration of spatial and temporal information during floral induction in Arabidopsis. Science 309: 1056-1059.

4. Liu C, Chen H, Er HL, Soo HM, Kumar PP, et al. (2008) Direct interaction of AGL24 and SOC1 integrates flowering signals in Arabidopsis. Development 135: 1481-1491.

5. Yoo SK, Chung KS, Kim J, Lee JH, Hong SM, et al. (2005) CONSTANS activates SUPPRESSOR OF OVEREXPRESSION OF CONSTANS 1 through FLOWERING LOCUS T to promote flowering in Arabidopsis. Plant Physiol 139: 770-778.

6. Immink RG, Pose D, Ferrario S, Ott F, Kaufmann K, et al. (2012) Characterization of SOC1's central role in flowering by the identification of its upstream and downstream regulators. Plant Physiol 160: 433-449.

7. Michaels SD, Ditta G, Gustafson-Brown C, Pelaz S, Yanofsky M, et al. (2003) AGL24 acts as a promoter of flowering in Arabidopsis and is positively regulated by vernalization. Plant J 33: 867-874.

8. Abe M, Kobayashi Y, Yamamoto S, Daimon Y, Yamaguchi A, et al. (2005) FD, a bZIP protein mediating signals from the floral pathway integrator FT at the shoot apex. Science 309: 1052-1056.

9. Hartmann U, Hohmann S, Nettesheim K, Wisman E, Saedler H, et al. (2000) Molecular cloning of SVP: a negative regulator of the floral transition in Arabidopsis. Plant J 21: 351-360.

10. Li D, Liu C, Shen L, Wu Y, Chen H, et al. (2008) A repressor complex governs the integration of flowering signals in Arabidopsis. Dev Cell 15: 110-120.

11. Deng W, Ying H, Helliwell CA, Taylor JM, Peacock WJ, et al. (2011) FLOWERING LOCUS C (FLC) regulates development pathways throughout the life cycle of Arabidopsis. Proc Natl Acad Sci U S A 108: 6680-6685.

12. Lee J, Oh M, Park H, Lee I (2008) SOC1 translocated to the nucleus by interaction with AGL24 directly regulates leafy. Plant J 55: 832-843.

13. Moon J, Lee H, Kim M, Lee I (2005) Analysis of flowering pathway integrators in Arabidopsis. Plant Cell Physiol 46: 292-299.

14. Kaufmann K, Wellmer F, Muino JM, Ferrier T, Wuest SE, et al. (2010) Orchestration of floral initiation by APETALA1. Science 328: 85-89.

15. Jang S, Torti S, Coupland G (2009) Genetic and spatial interactions between FT, TSF and SVP during the early stages of floral induction in Arabidopsis. Plant J 60: 614-625.

16. Helliwell CA, Wood CC, Robertson M, James Peacock W, Dennis ES (2006) The Arabidopsis FLC protein interacts directly in vivo with SOC1 and FT chromatin and is part of a high-molecular-weight protein complex. Plant J 46: 183-192.

17. Jaeger KE, Pullen N, Lamzin S, Morris RJ, Wigge PA (2013) Interlocking feedback loops govern the dynamic behavior of the floral transition in Arabidopsis. Plant Cell 25: 820-833.

18. Wagner D, Sablowski RW, Meyerowitz EM (1999) Transcriptional activation of APETALA1 by LEAFY. Science 285: 582-584.

19. Cavelier G, Anastassiou D (2004) Data-based model and parameter evaluation in dynamic transcriptional regulatory networks. Proteins 55: 339-350.

20. Buchler NE, Louis M (2008) Molecular titration and ultrasensitivity in regulatory networks. J Mol Biol 384: 1106-1119.
